# Editorial: Placebo Effect in Pain and Pain Treatment

**DOI:** 10.3389/fpain.2022.884055

**Published:** 2022-03-29

**Authors:** Michael H. Bernstein, Charlotte Blease, Lene Vase

**Affiliations:** ^1^Department of Behavioral and Social Sciences, Center for Alcohol and Addiction Studies, School of Public Health, Brown University, Providence, RI, United States; ^2^Division of General Medicine, Beth Israel Deaconess Medical Center, Boston, MA, United States; ^3^Department of Psychology and Behavioral Science, Division for Psychology and Neuroscience, Aarhus University, Aarhus, Denmark

**Keywords:** placebo & nocebo effects, pain, pain management, analgesia, placebo response

## Why We Need Pain Research: Chronic Pain, COVID-19, and the Opioid Crisis

Research into the biology, treatment, and prevention of pain has blossomed over the past decade. Recent data from the US suggest the nationwide prevalence of chronic pain is 20–21% (1, 2). Chronic pain appears more prevalent among women and older adults (3). A meta-analysis indicates that the prevalence of chronic pain in the UK is 35–51%. Cohen et al. (4) argue “It is difficult to over-estimate the burden of chronic pain,” (p. 2082) given the considerable price we pay in terms of economic cost, disability, and mortality.

Unfortunately, the public health burden of chronic pain will likely increase with the COVID-19 pandemic. Research suggests that 10–30% of people who contract COVID-19 develop Long Covid (5, 6). Pain is a common Long COVID symptom; in one study of 616 adults who self-reported a prior COVID-19 diagnosis, 30.7% met criteria for Fibromyalgia (7). As of 22 February 2022, ~428 million people world-wide have had COVID-19 (according to https://www.kff.org/coronavirus-covid-19/issue-brief/global-covid-19-tracker/). Using conservative projections based on the above statistics, if 10% of COVID-19 patients have long COVID, and 15% of long COVID patients develop chronic pain, then COVID has thus far coincided with an increase of 6.4 million chronic pain patients. This number will continue to grow with future infections.

The crisis of opioid misuse and death intersects heavily with pain management (8). In 2015–2016 ~50,000 people died annually in the U.S. from a drug overdose (9). This figure has since almost doubled (10). A November 17 front-page story in *The New York Times* reads: “Overdose Deaths Reached Record High as the Pandemic Spread.” It is imperative that we invest in research to understand the etiology of pain and develop effective pain treatments.

## Pain and the Placebo Effect

A wide and tantalizing body of research dating back to the mid-1950s has suggested that placebo may play an important role in pain treatment (11, 12). In randomized trials, patients improve on placebo for a variety of pain conditions (13). Although controversial (14, 15), this finding indirectly suggests placebo effects administered via placebos promote analgesia. Disentangling the placebo effect from other factors (e.g., assessment reactivity, passage of time) is critical to how we interpret Randomized Clinical Trials (RCTs) (16, 17). The placebo effect also features prominently in studies investigating analgesia from pain induced in the laboratory (18). Painkillers such as morphine are more effective when a patient is aware, vs. unaware, they are receiving the drug (19), which demonstrates the psychological nature of placebo analgesia. But the placebo effect is physiological as well. The endogenous opioid, endoconnabinoid, and dopaminergic systems are all implicated in placebo pain-relief (20).

## Special Issue Articles

The articles that follow show the diversity of topics related to the placebo effect and pain. Wampold and Smith et al. remind us that the placebo effect is often conceptualized broadly to include interpersonal factors (21) [see (22) for a lengthy discussion of placebo definitions]. Wampold situates the placebo effect within a healing context and argues for the importance of evolutionary considerations in how humans (and non-humans) improve through interpersonal contact. Smith et al. leverage the importance of empathy and optimism by describing a training program for primary care physicians, called *Empathico*, to enhance communication skills and ultimately reduce patient suffering.

Two experimental studies are published in this Special Topic. Wagner et al. observe that neither a dog nor a placebo increases pain tolerance among healthy volunteers in the lab. This paper serves as an important reminder about the need to publish non-significant results to mitigate the chance that we see a replication crisis within placebo studies, as has been observed across other scientific disciplines including the adjacent field of psychology (23). The paper by Lunde et al. further illustrates the importance of developing adequate placebo control conditions for non-pharmacological interventions like music analgesia. By introducing a placebo control to music analgesia, it is specified that the analgesic effect of music primarily steams from patients expectations rather than music *per se*.

With respect to placebo control arms in drug treatment trials, Koechlin et al. examined the placebo response to anti-depressant medication for Fibromyalgia in a meta-analysis of randomized trials. Pain, functional disability, and depression were all reported to have improved among patients taking a placebo. Again, disentangling the placebo effect from other confounding factors would be an invaluable follow-up should a no-treatment arm be deemed ethical.

Turning away from double-blind placebo, a growing number of studies suggest placebos can be effective even when given openly (24) (so-called Open-Label Placebo [OLP]), though some have raised methodological limitations with this line of research (15, 25). Four studies explore the topic of OLP. Estudillo-Guerra et al. report an interesting case study from a prior pilot OLP study (26) where a patient was successfully able to taper off oxycodone using a placebo conditioning paradigm without experiencing an increase in pain. Leveraging placebos to reduce opioid use is one of the most important translational aspects of placebo research (27) with some initial evidence of efficacy (26, 28). Sezer et al. report a pre-registration of another such trial where patients are randomized to Treatment as Usual (TAU) or TAU plus four OLP injections for post-operative pain. This will be the first randomized trial of OLP we are aware of to examine the efficacy of an honest placebo delivered intravenously. Heiss et al. argue that we re-examine the rationale provided in OLP studies by suggesting two new rationales that may be more effective than the standard rationale (29) for some patients. Finally, Wang et al., building off of the finding that there is a placebo genome [named the “placebome” (30)] show that there are genetic markers which can predict the placebo to honest placebo, namely being homozygous for rs4680, which is a single nucleotide polymorphism incatechol-O-methyltransferase.

## Conclusion

With the ongoing opioid crisis and COVID-19 pandemic, chronic pain has become an especially serious public health concern. As the articles in this special issue demonstrate, understanding and leveraging the placebo effect may play an important role in addressing pain.

## Author Contributions

MB wrote the initial draft. All authors make corrections and edited the manuscript. All authors contributed to the article and approved the submitted version.

## Funding

MB was supported by K01DA048087.

## Conflict of Interest

The authors declare that the research was conducted in the absence of any commercial or financial relationships that could be construed as a potential conflict of interest.

## Publisher's Note

All claims expressed in this article are solely those of the authors and do not necessarily represent those of their affiliated organizations, or those of the publisher, the editors and the reviewers. Any product that may be evaluated in this article, or claim that may be made by its manufacturer, is not guaranteed or endorsed by the publisher.
